# Formulation development, characterization, and optimization of Eudragit–cinnamon essential oil-based nanoplatform and its efficacy against resistant bacteria

**DOI:** 10.3389/fchem.2025.1555449

**Published:** 2025-08-08

**Authors:** Touseef Nawaz, Niamat Ullah, Muhammad Ali, Safia Obaidur Rab, Irfan Ahmad, Rabia Baloch, Sadia Chaman, Kifayat Ullah Shah, Adnan Amin

**Affiliations:** ^1^ Department of Pharmaceutics, Faculty of Pharmacy, Gomal University, Dera Ismail Khan, Pakistan; ^2^ NPRL, Department of Pharmacognosy, Faculty of Pharmacy, Gomal University, Dera Ismail Khan, Pakistan; ^3^ Central Labs, King Khalid University, Abha, Saudi Arabia; ^4^ Department of Clinical Laboratory Sciences, College of Applied Medical Sciences, King Khalid University, Abha, Saudi Arabia; ^5^ Allama Iqbal Teaching Hospital, Dera Ghazi Khan, Pakistan; ^6^ Institute of Pharmaceutical Sciences University of Veterinary and Animal Sciences Out Fall Road UVAS, Lahore, Pakistan; ^7^ Department of Life Sciences, Yeungnam University, Gyeongsan, Republic of Korea

**Keywords:** Calibri bacterial resistance, Design-Expert, essential oil, Eudragit, nanoplatform

## Abstract

**Introduction:**

Microbial resistance is a growing global concern, necessitating the development of novel drug delivery system to combat the resistant bacterial strains. We aimed to formulate Eudragit based cinnamon essential oil loaded nanoplatform against resistant microbial strain.

**Methods:**

Nanoparticles were characterized for zeta potential, PDI, particle size, SEM, FTIR, entrapment efficiency and drug release kinetic. Box Behnken design with the quadratic model was used to check the effect of independent factors and dependent factors.

**Results and Discussion:**

The *Klebsiella* and *Staphylococcus* aureus have shown same MIC value of 1.25 μL/ml while *E. coli* and *Pseudomonas aeruginosa* shown 0.078 and 0.625 μL/ml respectively. Quadratic polynomial equation depicted that stirring speed exhibited negative effect on the PDI, particle size and encapsulation efficiency. The polymer concentration produced positive effect on the particle size, PDI and encapsulation efficiency of the nanoparticles. The predicted response values were as particle size (Y1) 228.9 nm, PDI (Y2) 0.3 and %EE (Y3) 72.75% which were very close to the actual values of response as particle size (Y1) was 230.4 ± 3.46 nm, PDI (Y2) was 0.293 ± 0.022, and %EE (Y3) was 74.9 ± 2.32%. It was concluded that our prepared formulation can be effectively used treat resistant bacterial infections.

## 1 Introduction

Antibiotic resistance occurs when pathogens evolve to resist treatment, increasing the spread of disease, chronic illness, and mortality (Chinemerem Nwobodo et al., 2022). It poses a significant threat to healthcare systems (Sharma, 2021), with projections indicating that by 2050, resistant microorganisms could cause 10 million deaths annually and cost the global economy £64 trillion (Baekkeskov et al., 2020; Dadgostar, 2019). As drug resistance grows and existing antibiotics lose effectiveness, the economic and mortality burdens rise (Saha and Sarkar, 2021). Therefore, there is an urgent need for the discovery and development of novel antimicrobial alternatives from herbal sources.

Nanoparticles have gained significant attention for their small size, sustained action, large surface area, and targeted drug delivery capabilities (Yusuf et al., 2023). They address common issues such as instability, low entrapment efficiency, and drug leakage, making them more efficient in various applications (Kumar et al., 2023). Consequently, their use is rapidly growing across industries including pharmaceuticals, food, agriculture, and cosmetics (Malik et al., 2023).

In the domain of drug delivery systems, different polymers have proven themselves essential for creating nanoplatforms. Biomaterials are classified as biodegradable and non-biodegradable. After discovering these materials, fields like controlled drug delivery, gene therapy, regenerative medicine, and tissue engineering have been revolutionized (Trucillo, 2024). These biomaterials are available in both natural and synthetic forms. Polysaccharides and proteins are natural biopolymers, and poly(meth)acrylate, polycaprolactone, poly(glutamic) acid, etc., are synthetic biopolymers. Synthetic biopolymers have advantages over natural biopolymers, like minimum immunogenicity and ease of modification for specific functions, making synthetic biopolymers more valuable in advanced medical applications (Satchanska et al., 2024). Poly(meth)acrylates are commercially known as Eudragit, a cationic polymer that is synthesized by the polymerization of acrylic and methacrylic acids or their ester (Dos Santos et al., 2021). It has poor aqueous solubility and also used as a film coating material, has sustained release, and has been used in drug delivery applications for the colon, vagina, ophthalmic, transdermal, enteric, gene and vaccine delivery, etc. Eudragit polymers with different grades and properties can be combined with other polymers to develop a novel blend that has the ability to modify the drug release at the target site and improve stability by film coating the formulation (Ullah et al., 2021). Previous studies reported that Eudragit effectively encapsulates and ensures protection of the biological activities of essential oil (Lee and Park, 2015; Mohsen et al., 2023).

Cinnamon essential oil (EO) is used in food preservation (Kacaniova et al., 2021), packaging (Niu et al., 2018), herbal pharmaceuticals (El Atki et al., 2019), and perfumes (Spence, 2024). It contains active compounds like cinnamaldehyde, which is effective in treating various diseases and has been traditionally used for toothaches, urinary tract infections, and stomach irritation (Knauth et al., 2018). Additionally, cinnamon EO is a strong antimicrobial agent (Liao et al., 2021). However, its pharmaceutical use is limited due to chemical instability, volatility, and poor solubility (Weisany et al., 2022). These challenges can be addressed by encapsulating cinnamon EO using methods like emulsification (Dghais et al., 2023), ionic gelation (Subasinghe and Wickramarachchi, 2019), and nanoprecipitation (Lammari et al., 2020). This polymeric encapsulation via nanoprecipitation offers targeted delivery, prolonged retention, and better therapeutic outcomes. This study thus focused on preparing and evaluating a cinnamon EO-loaded Eudragit nanoplatform that would be effective against resistant bacterial strains.

## 2 Methodology

### 2.1 Chemicals, polymers, growth media, and bacterial strains

The chemicals, including polyvinyl alcohol and resazurin (Sigma Aldrich, St. Louis, MO, United States), were purchased commercially, whereas Eudragit L-100 was generously donated by BioLab Islamabad. Cinnamon essential oil extracted in the lab (NPRL) was used as the surfactant (Sigma Aldrich). The bacterial growth media, including nutrient agar (Hi Media, Mumbai, India), tryptic soy broth (Hi Media, Mumbai, India), and Luria–Bertani Broth (LB) (Oxoid, Hampshire, United Kingdom), were obtained commercially. The resistant bacterial strains *E. coli, Staphylococcus aureus, Pseudomonas aeruginosa*, and *Klebsiella pneumoniae* were obtained from NPRL, Gomal University, D.I.Khan.

### 2.2 Essential oil extraction

Cinnamon essential oil was extracted through a hydro distillation method using a Clevenger apparatus with slight modification, as mentioned by Imran et al. (2024). Briefly, cinnamon bark was purchased from the local market (D.I.Khan, KPK, Pakistan). Hundred grams of cinnamon bark was ground and fed into the Clevenger apparatus along with distilled water. In order to extract the essential oil, this operation was carried out for up to 8 h, and after that, the extracted essential oil was separated.

### 2.3 Preparation of cinnamon EO oil-loaded nanoparticles

Eudragit-based nanoparticles loaded with cinnamon EO were prepared via a standard nanoprecipitation protocol already reported by Jummes et al. (2020). Eudragit was dissolved in methanol, and 200 µL of essential oil was mixed with the polymeric solution. After that, the essential loaded polymeric solution was added dropwise to the surfactant (polyvinyl alcohol) solution with constant stirring. The nanoparticle suspension was stirred for 3 h to completely evaporate the organic solvent.

### 2.4 Optimization of experimental design

Optimization is a robust approach for developing a precise and accurate formulation with all the properties of an ideal formulation across all aspects. In this study, Design-Expert software (Design-Expert 13^®^, State Ease Inc., United States) was used to optimize formulation by employing a Box–Behnken design. Three independent variables: stirring speed, surfactant concentration, and polymer concentration, and three dependent variables: particle size, polydispersity index (PDI), and Entrapment Efficiency (%EE) were evaluated for formulation optimization. As outlined in a table (Tables 1, 2), a total of 16 experimental runs were performed for optimal formulation optimization, with inclusion of statistical tools to compare the predicted versus actual values. The selected optimized formulation was subjected to further physicochemical characterization. The final optimized formulation was selected on the basis of desirability factors in relation to the response variables.

**TABLE 1:** Factors and responses with formulation parameters.

S.No	Independent variables (factors)
X1	Stirring speed (rpm)
X2	Surfactant concentration (%)
X3	Polymer concentration (gm)
	Dependent variables (responses)
Y1	Particle size (nm)
Y2	PDI (%)
Y3	Entrapment efficiency (%)
	Formulation parameters that were kept constant
Z1	Methanol 15 mL
Z2	Oil concentration 200 μL
Z3	Total volume 55 mL
	Level of significance (α) 0.05

**TABLE 2:** Designing independent variables, three factors at three levels with coded and actual values.

Factor levels	Coded values	Actual values		
		Stirring speed (X1)	Surfactant concentration (X2)	Polymer concentration (X3)
Low	−1	300	0.5	200
Medium	0	600	1	400
High	+1	900	1.5	600

### 2.5 Particle size, zeta potential, and polydispersity index

Zeta potential, hydrodynamic particle size, and polydispersity index were determined through a zeta sizer with a standard protocol with slight modification, as reported by Ruiz et al. (2022). Briefly, the average hydrodynamic diameter (z-Average) was obtained by dynamic light scattering. A nanoparticle suspension was diluted 50% with deionized water and fed into the zeta sizer (Malvern Instruments Ltd., United Kingdom). The same dilution was used for determining the ζ-potential and PDI.

### 2.6 Encapsulation efficiency

Encapsulation efficiency of essential oil-loaded Eudragit nanoparticle was determined using the method reported by Jummes et al. (2020) with a slight modification. The nanoparticle suspension was centrifuged at 6,000 rpm for 20 min to separate the encapsulated essential oil from the free essential oil. The supernatant solution was analyzed for free essential oil using UV spectroscopy. Encapsulation efficiency was determined using the following equation:

$$\%EE=\frac{Initial\;EO-Free\;EO}{Initial\;EO}\times 100.$$

### 2.7 *In vitro* drug release study

The *in vitro* drug release study was accomplished using the dialysis bag diffusion method, known for its widespread use and flexibility in assessing drug release from nanoplatforms. Concisely, a dialysis bag (MW 12000–14000, Sigma Co, NY, United States) was filled with a 1-mL aliquot of cinnamon-loaded Eudragit nanoparticles, and the ends of the bag were sealed securely using clippers. The bag was immersed in a phosphate buffer (pH 7.4) of 100 mL, and a stirring speed of 100 rpm and a temperature of 32 ± 1°C were maintained during an experiment to mimic skin *in vivo* conditions. Samples were withdrawn at 1 h, 1.5 h, 2 h, 4 h, 6 h, 8 h, 10 h, and 12 h and evaluated spectrophotometrically at 290 nm (Ullah et al., 2023). Fresh PBS was added in equivalent volumes to maintain consistency. The % cumulative release of cinnamon was calculated in triplicate. The trapezoidal rule was used to measure the release efficiency (% R.E) from the area under the curve at a given time point. % R.E was calculated using the following equation.

$$\%\;R.E=\frac{\int_{0}^{t}Y\times \mathrm{d}t}{Y_{t}\times 10}\times 100,$$

where Y = % drug release.

T = time at which % drug is released.

Various mathematical models, including zero-order, first-order, Peppas, and Higuchi models, were used to elucidate the drug release mechanism of cinnamon-loaded Eudragit nanoparticles. Linear regression analysis was performed to find the fitness of each model, and the regression coefficient (*R* ^2^) was determined to find the suitable model. The model with an *R* ^2^ value close to 1 implied the best fit. According to the Peppas theory, if the release exponent (n) is smaller than or equal to 0.43, it will follow the Fickian diffusion method. If the n value is greater than 0.43 and smaller than 0.85, the release will follow a non-Fickian or anomalous diffusion process. If the n value is 0.85, it will follow case II transport mechanism, while n greater than 0.85 will follow super case II transport, which is a more complex release mechanism.

### 2.8 FTIR

FTIR was performed to check the interaction between the active and inactive ingredients of the formulation. The spectra of the cinnamon essential oil, Eudragit, and the cinnamon essential oil-loaded Eudragit-based nanoparticle formulation were determined using ATR-FTIR fitted with an ATR sampling cell (Thermo Scientific, Waltham, MA, United States). A resolution of 4 cm^−1^ was used to obtain FTIR spectra between 4,000 cm^−1^ and 400 cm^−1^. Two scans of each sample were performed, and spectra were obtained using OPUS 9 software (Nawaz et al., 2021).

### 2.9 Surface morphology

The optimized cinnamon-loaded Eudragit nanoparticles were meticulously evaluated for morphological characterization using a scanning electron microscope (SEM). Prior to SEM analysis, the optimized nanoparticles underwent lyophilization using a freeze dryer (Biobase, Shandong, China). After lyophilization, the optimized cinnamon-loaded Eudragit nanoparticles were carefully mounted onto aluminum stubs and affixed securely using adhesive carbon tape to prevent contamination and particle displacement during analysis. The SEM (Carl Zeiss Inc., Oberkochen, Germany) was operated at an accelerated voltage of 10 KV under high vacuum conditions to capture high-resolution and detailed images. This setup precisely visualizes the morphological characteristics important for understanding the structural and surface qualities of the optimized cinnamon-loaded Eudragit nanoparticles (Hadidi et al., 2020).

### 2.10 Biological evaluation of essential oil

#### 2.10.1 Inhibition zones (mm)

The zone of inhibition of the cinnamon EO against the bacterial strain was determined through a standard protocol of the disk diffusion method already reported by Al Zuhairi et al. (2020). Briefly, a 10% solution of the cinnamon EO was prepared in DMSO. Then, Mueller–Hinton agar plates were prepared, and a 24-h-old bacterial strain culture matched with 0.5 McFarland turbidity was spread on the plates. A 6-mm disk was put in the center of the plate and soaked with the already prepared 10% solution of the essential oil (10 µL). The plates were then incubated at 37°C for 24 h. Finally, the zone of inhibition was measured.

#### 2.10.2 Minimum inhibitory concentration (MICs)

The minimum inhibitory concentration of the cinnamon EO was determined by the 96-well microplate method with slight modification (Chakansin et al., 2022). Briefly, 50 µL of the nutrient broth was poured into all the wells of the 96-well microplate. After that, 50 µL of the test sample was loaded into the very top well and diluted serially downward. Finally, a 24 h-old bacterial culture (50 µL) was added into each well and incubated for 24 h. The same procedure was adopted for the control with the test sample. After the incubation period, resazurin (0.015%, 10 µL) was added to each well and incubated further for 1 hr. Afterward, the colorimetric method was used to determine MICs.

## 3 Results and discussion

### 3.1 Optimization of cinnamon-loaded Eudragit nanoparticles

The optimization of cinnamon-loaded Eudragit nanoparticles was performed using a Box–Behnken design to systematically evaluate the effect of factors (independent variables) on various selected responses (dependent variables). The approach involved using 3D surface plots and contour plots to conceive the impact of factors. The interconnection between the variables and responses was mathematically quantified using the polynomial equation, which apprehends the factors’ individual and combined effects on each response. The quadratic model was considered more suitable for all independent variables due to its capacity to maximize the impact on individual and combined analysis. The maximum and minimum levels of material selection were based on prior knowledge of the experimental system and practical constraints. The minimum level (−1) was typically the lowest feasible value of a factor, often representing a baseline or lower experimental condition, while the maximum level (+1) was the highest feasible value, representing the upper bound of the factor’s range. These levels were chosen to explore the factor’s influence effectively within realistic experimental limits. The selected levels ensured meaningful exploration of the system while adhering to practical constraints, including equipment limits and safety. Design-Expert software aided this statistical analysis, including ANOVA to ratify the model fitting for experimental data. Table 3 shows the results in detail for the two-factor interaction (2FI), linear, and quadratic models.

**TABLE 3:** Box–Behnken design-based design of experimentation for 16 trial formulations.

Runs	X1	X2	X3	Y1	Y2	Y3
F1	300	0.5	400	418.7	0.433	80
F2	300	1	200	198.6	0.275	68.3
F3	300	1	600	718.7	0.499	85
F4	300	1.5	400	331.9	0.367	78.5
F5	600	0.5	200	310.2	0.332	77
F6	600	0.5	600	511.6	0.483	83
F7	600	1	400	227.6	0.300	72
F8	600	1	400	234.5	0.291	75
F9	600	1	400	221.9	0.312	71
F10	600	1	400	231.5	0.297	73
F11	600	1.5	200	332.5	0.376	79.8
F12	600	1.5	600	457.1	0.422	82
F13	900	0.5	400	209	0.278	69
F14	900	1	200	190.6	0.212	66.4
F15	900	1	600	227.6	0.3	70
F16	900	1.5	400	268	0.345	75.3

X1, stirring speed (RPM); X2, surfactant concentration (%); X3, polymer concentration (mg); Y1, particle size (nm); Y2, PDI; Y3, entrapment efficiency (%).

Key formulation parameters, such as stirring speed (X1), surfactant concentration (X2), and polymer concentration (X3), were evaluated at three levels: −1 for low, 0 for medium, and 1 for high, for the optimization. The selection of the optimized formulation was based on accomplishing specified criteria, chiefly the smallest particle size, low PDI, and high entrapment efficiency, as analyzed by Design-Expert.

A total of 16 formulations with four central points were analyzed. The smallest particle size of 190.6 nm was ascertained in formulation F14, while the largest particle size of 718.7 nm was observed for formulation F3. The lowest PDI of 0.212 was demonstrated by formulation F14, while formulation F3 showed the highest PDI of 0.499. Formulation F3 showed a %EE of 85%, while formulation F14 demonstrated the lowest %EE of 66.4%. The highest coefficient of determination (*R* ^*2*^) value, between 0.9585 and 0.9333, demonstrates a strong correlation between model-predicted and experimental data. Furthermore, the high Predicted Residual Error Sum of Squares (PRESS) value validates the model robustness, as summarized in Table 4.

**TABLE 4:** Regression analysis summary for various data fitting models.

Model	*R* ^2^	Adjusted *R* ^2^	SD	Mean	% CV	p-value
Response Y1						
Quadratic	0.9585	0.8952	46.77	318.10	14.70	0.0018
Linear	0.5513	0.4392	108.17	318.10	34.01	0.0187
2FI	0.7598	0.5996	91.40	318.10	28.73	0.0189
Response Y2						
Quadratic	0.9684	0.9210	0.022	0.3460	6.45	0.0008
Linear	0.6017	0.5021	0.056	0.3460	16.18	0.0095
2FI	0.7171	0.5286	0.054	0.3460	15.74	0.0361
Response Y3						
Quadratic	0.9333	0.8331	2.30	75.39	3.05	0.0067
Linear	0.4792	0.3489	4.54	75.39	6.02	0.0435
2FI	0.5959	0.3265	4.62	75.39	6.12	0.1369

Y1, particle size; Y2, PDI; Y3, entrapment efficiency; *R* ^2^, coefficient of correlation; CV, coefficient of variation.

### 3.2 Effect of independent factors on particle size (Y1)

**FIGURE 1:**
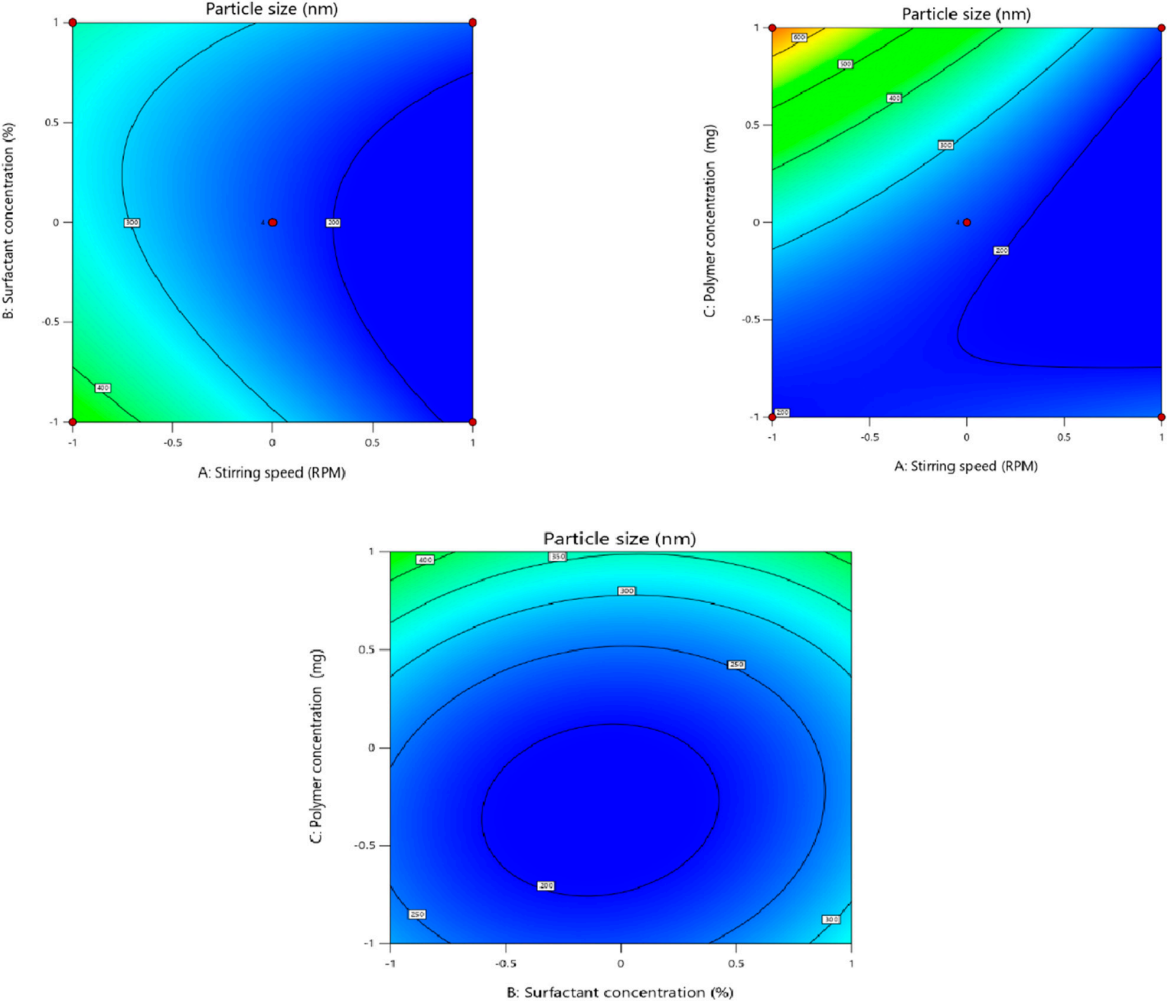
Effect of independent variables (stirring speed, polymer concentration, and surfactant concentration) on the dependent variable (particle size).

**FIGURE 2:**
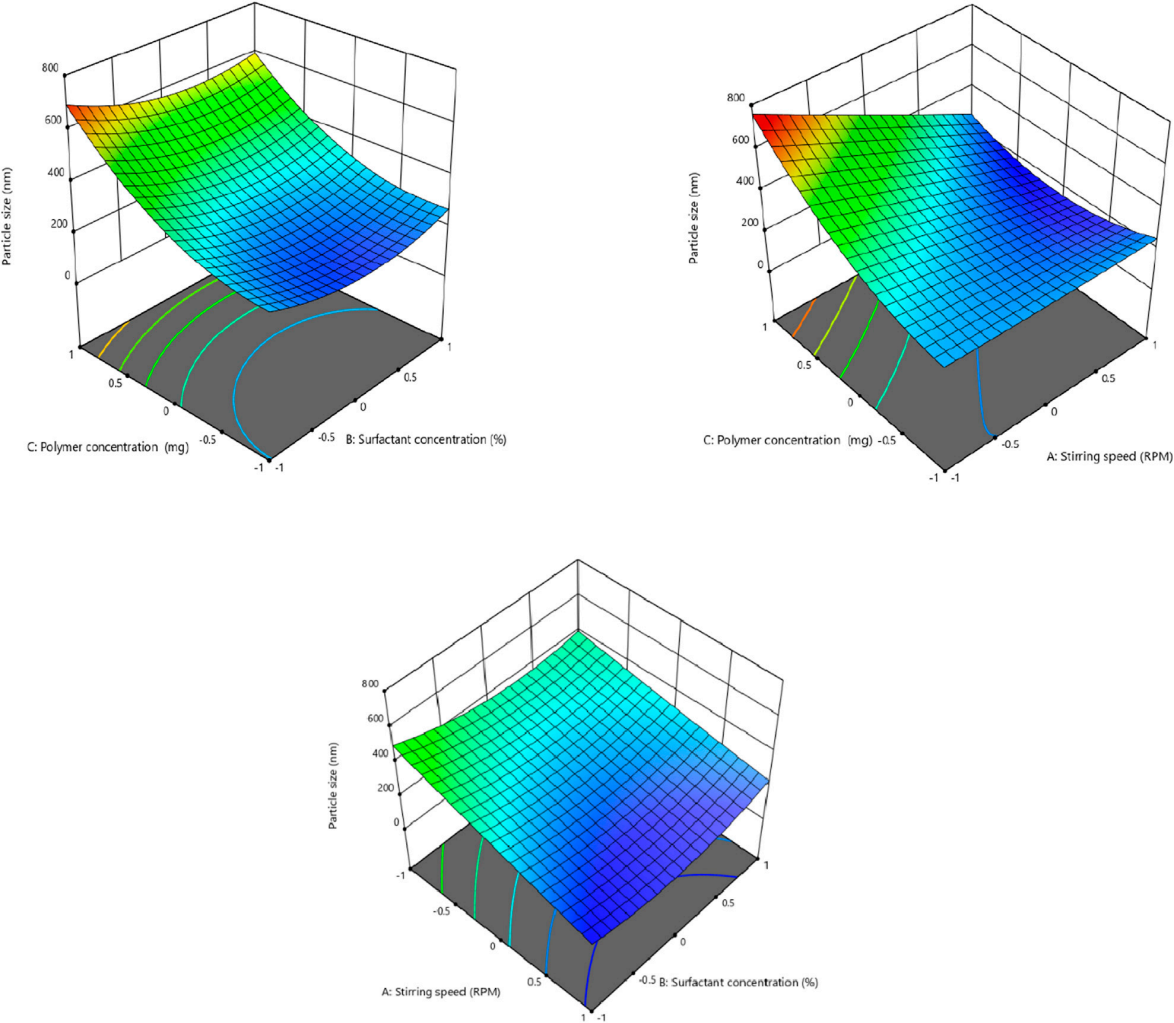
3D presentation of the effect of independent factors on the particle size.

In order to fit the response with the experimental data and create a second-order polynomial model as given by the equation below, the size (Y1) (dependent variable) obtained at three distinct independent variables (X1, X2, and X3), respectively, as presented in the model, was subjected to multiple regression analysis. The effects of stirring speed (X1), surfactant concentration (X2), and polymer concentration (X3) effect were checked on dependent variables like particle size. Particle size ranged from 190.6 nm to 718.8 nm. It was observed that the stirring speed exhibited a negative effect on the particle size. Surfactant concentration has a negative effect on particle size at the medium concentration and a positive effect on particle size in low and high concentrations. Eudragit (polymer) concentration had a positive impact on the particle size of the nanoparticles. Moreover, it is clear from the polynomial equation that stirring speed and surfactant concentration (X1X2) have a synergistic effect on the particle size, keeping the polymer concentration constant (Figures 1, 2).

Particle size (Y1) = +228.87–96.64 X1-7.50 X2+110.34X3+36.45 X1X2-120.87 X1X3-19.20 X 2 X 3+4.48 X1^2^ + 73.55 X2^2^ + 100.43 X3^2^

The regression coefficients for the linear, square, and interaction effects are represented by β1 to β9 in Table 5, whereas β◦ denotes the intercept with the y-axis. In the equation, an *R* ^2^ value of 0.95 showed that the experimental and projected data had a strong correlation with one another. A *R* ^2^ value of 0.50 or above was considered reasonable, as reported previously (Siagian et al., 2021). The p-values less than 0.05 were considered significant (Di Leo and Sardanelli, 2020) so in this case, X1, X3, X1X3, X2^2^, and X3^2^ are the significant model terms (Table 5).

**TABLE 5:** Summary of regression coefficient analysis for response Y1 (particle size).

Coefficients	β0	β1 (X1)	β2 (X2)	β3 (X3)	β4 (X1X2)	β5 (X1X3)	β6 (X2X3)	β7 (X1^2^)	β8 (X2^2)^	β9 (X3^2^)
Size (nm)	228.87	−96.64	−7.50	110.34	36.45	−120.87	−19.20	4.48	73.55	100.43
p-value	0.0018	0.0011	0.6661	0.0005	0.1701	0.0021	0.4430	0.8545	0.0199	0.0051

Previous literature revealed that the polymer concentration positively influences the particle size of the nanoparticles (Elmowafy et al., 2021). This tendency, which causes nanoparticles to destabilize and agglomerate, is caused by increased viscous forces that hinder particle disintegration and interfere with appropriate emulsification and stability of nanoparticles (Kiparissides and Pladis, 2022). Surfactant concentration decreases the particle size of the nanoparticles at the optimum concentration. Decreasing or increasing the concentration causes aggregation of the particles, and ultimately, the particle size increases. This behavior is attributed to the fact that at higher concentrations of surfactant, particles aggregate due to the sticky nature of the surfactant, while at lower concentrations, less of the surfactant is available to coat the particles, so larger particles are formed (Routray et al., 2022). The stirring speed significantly influences the particle size of the nanoplatform, possibly because a higher stirring speed is related to a higher energy dissipation in the system (Schwaminger et al., 2020).

### 3.3 Effect of independent factors on PDI (Y2)

The polydispersity index (Y2) (dependent variable) obtained at three different independent variables, including polymer concentration, surfactant concentration, and stirring speed, was then further subjected to multiple regression analysis to fit the response with the experimental data and to produce a model of a second-order polynomial equation.

$$\text{PDI }\left(Y2\right)=0.3000-0.0531X1-0.0020X2+0.0654X3+0.0332X1X2-0.0305X1X3-0.0262X2X3-0.0113X1^{2}+0.0670X2^{2}+0.0363X3^{2}$$

**FIGURE 3:**
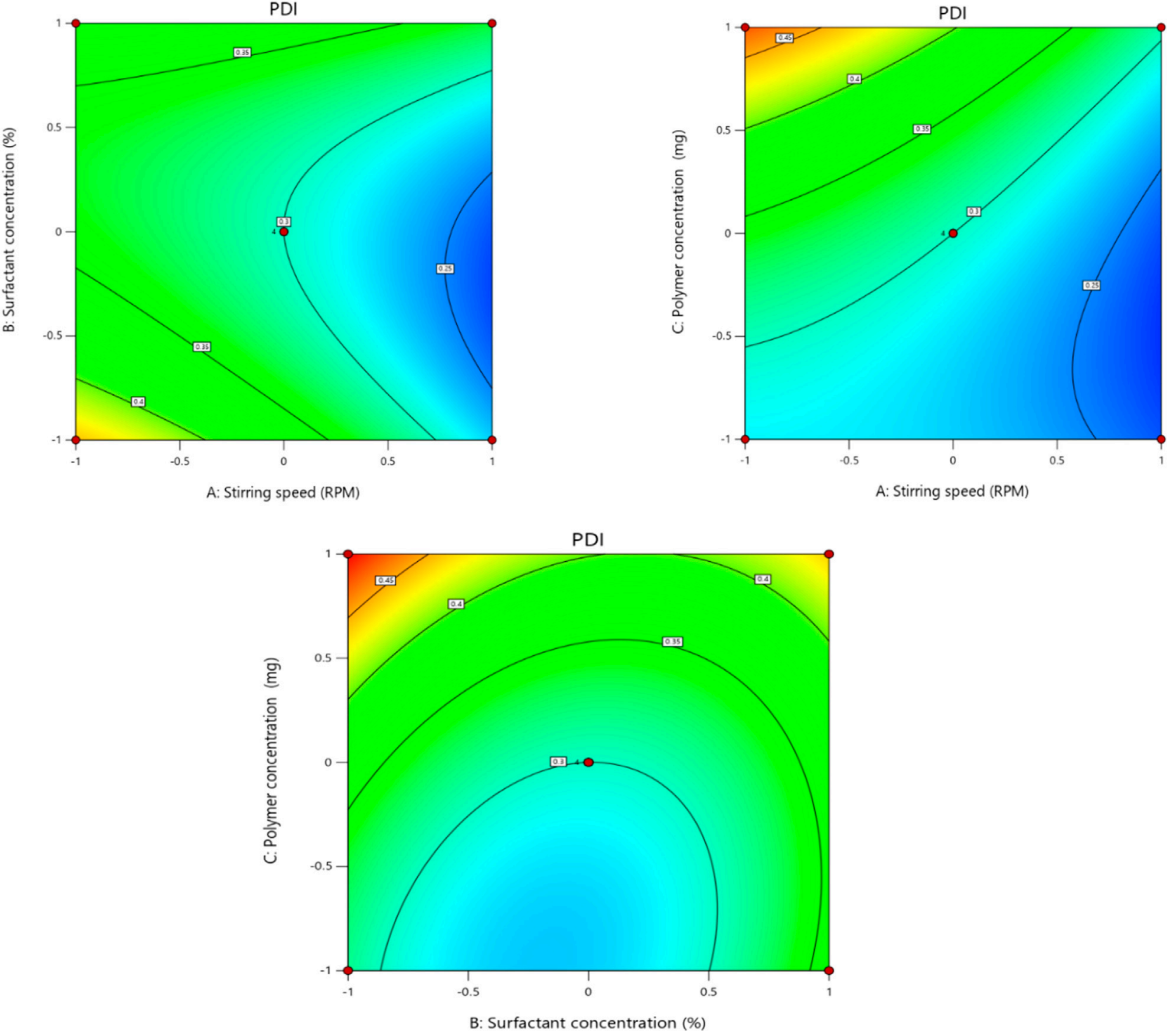
Effect of independent variables (stirring speed, polymer concentration, and surfactant concentration) on the dependent variable (PDI).

**FIGURE 4:**
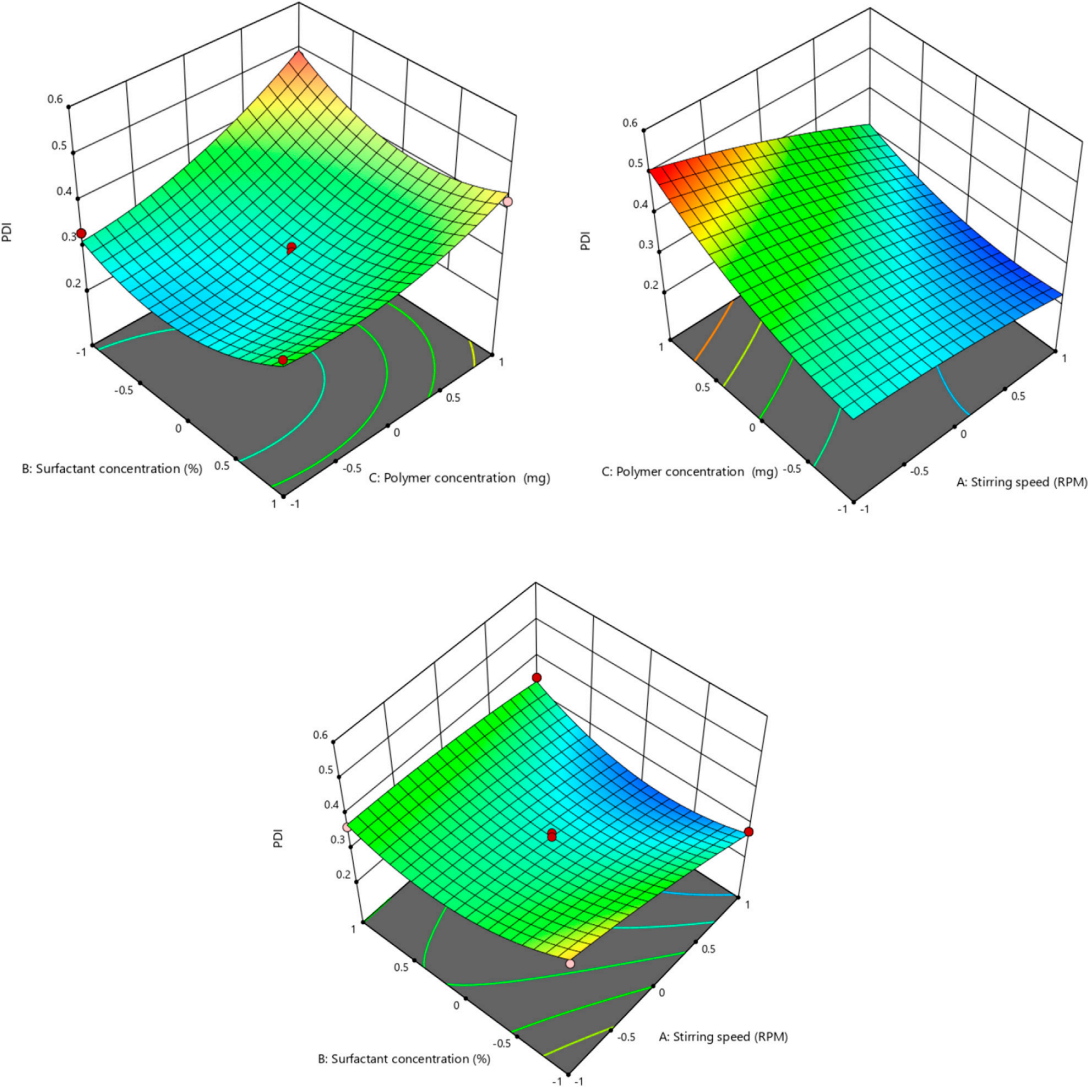
3D presentation of the effect of independent factors on PDI.

PDI (Y2) of the formulation is the measure of the heterogeneity, and PDI<0.3 is considered the optimum range, while values ranging from 0.5 to 1 are considered the acceptable range (Onugwu et al., 2022). The PDI values of different formulations ranged from 0.212 to 0.499, as shown in Table 5. The polynomial equation contour graph and 3D graph show that the stirring speed (X1) has a negative effect on PDI, as previously mentioned by Manisekaran et al. (2022). Surfactant concentration also exhibited a slight negative effect on PDI of the nanoparticles, while polymer concentration showed a positive effect on the PDI of the nanoparticles, as mentioned in the early literature by Nawaz et al. (2021) (Figures 3, 4). An *R* ^2^ value of 0.9684 in the equation indicated a significant correlation between the projected and experimental data. The regression analysis in Table 8 shows that the model terms having a significant effect on the PDI of the nanoparticles include X1, X3, X1X2, X1X3, X1^2^, and X3^2^, as the p-values of all the mentioned model terms are <0.05 (Table 6).

**TABLE 6:** Summary of regression coefficient analysis for response Y2 (PDI).

Coefficients	β0	Β1 (X1)	β2 (X2)	β3 (X3)	β4 (X1X2)	β5 (X1X3)	β6 (X2X3)	β7 (X1^2^)	β8 (X2^2)^	β9 (X3^2^)
PDI	0.3000	−0.053	−0.002	0.0654	0.0332	−0.0305	−0.026	0.011	0.0670	0.0363
p-value	0.0008	0.0005	0.8083	0.0002	0.0246	0.0340	0.0586	0.3520	0.0010	0.0175

### 3.4 Effect of independent factors on entrapment efficiency (Y3)

**FIGURE 5:**
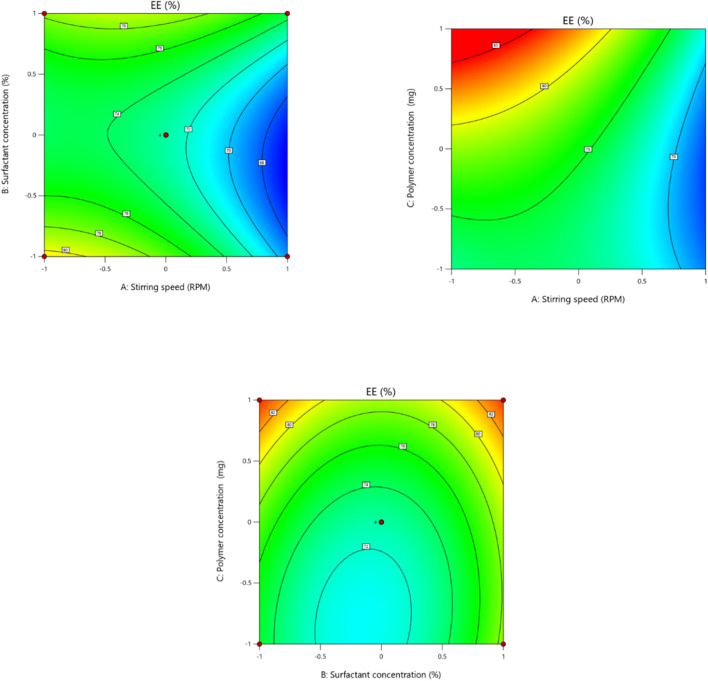
Effect of independent variables (stirring speed, polymer concentration, and surfactant concentration) on the dependent variable (EE).

**FIGURE 6:**
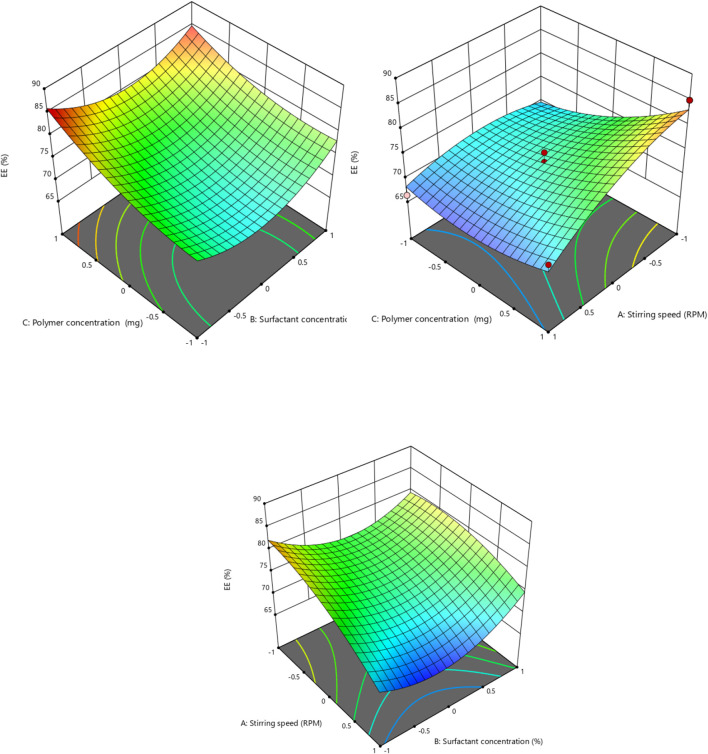
3D presentation of the effect of independent factors on EE.

Independent factor (stirring speed, surfactant concentration, and polymer concentration) effects were determined on the encapsulation efficiency (dependent variable) of the nanoparticle formulation. The encapsulation efficacies of the different formulations prepared range from 66.4% to 85%. According to the results of the ANOVA test, the linear model was significant and appropriate for the observed encapsulation efficiency % data. It was shown in the second-order polynomial equation that stirring speed (X1) has a negative effect on the encapsulation efficiency. Increasing the speed of stirring causes shear forces to increase, which may lead to the drug leaking out of the nanoparticles and ultimately lowering the entrapment efficiency of the nanoparticles (Bin-Jumah et al., 2020). Surfactant concentration has a slightly positive effect on the entrapment efficiency of the nanoparticle, as mentioned by Sharma et al. (2016) (Figures 5, 6). The terms of the model with a significant effect on the encapsulation efficiency of the nanoparticle include X1, X3, X1X2, X1X3, X22, and X32 (Table 7). The encapsulation technique not only reduces the evaporation rate of volatile components in EOs, but it also improves the antioxidant and antibacterial activity of these bioactives compared to their free forms as a result of protection against unfavorable effects such as oxygen and temperature (Hadidi et al., 2020). Hadidi and colleagues prepared cinnamon EO-loaded chitosan nanoparticles, and they observed that the encapsulation efficiency ranged from 55.8% to 73.4% (Hadidi et al., 2020).

**TABLE 7:** Summary of regression coefficient analysis for response Y3 (%EE).

Coefficients	β0	Β1 (X1)	β2 (X2)	β3 (X3)	β4 (X1X2)	β5 (X1X)	β6 (X2X3)	β7 (X1^2^)	β8 (X2^2)^	β9 (X3^2^)
%EE	72.75	−3.76	+0.8250	3.89	1.95	−3.03	0.950	−2.41	5.36	2.34
p-value	0.0008	0.0005	0.8083	0.0002	0.0246	0.0340	0.0586	0.3520	0.0010	0.0175

The retention rate of cinnamon essential oil (CEO) loaded into chitosan nanoparticles ranged from 55.8% to 73.4%, which was significantly (p < 0.05) affected by the ratio of chitosan/CEO.

$$\text{EE }\left(Y3\right)=+72.75-3.76\;X1+0.8250X2+3.69X3+1.95X1X2-3.03X1X3-0.9500X2X3-2.41X1^{2}+5.36\;X2^{2}+2.34X3^{2}$$

### 3.5 Preparation of optimized cinnamon EO-loaded Eudragit nanoparticles

The optimized cinnamon EO-loaded Eudragit nanoparticles were prepared using the nanoprecipitation method, which was utilized for all 16 trial formulations. The optimized formulation was selected based on parameters approved by Design-Expert^®^, which evaluated the outcomes of 16 trial formulations by considering the response. The optimized formulation was approved based on achieving a small particle size, low PDI, and high %EE. The final concentrations stated in the optimized formulation are the result of statistical prediction based on experimental data.

Design-Expert^®^ evaluated trial formulations. A formulation with a desirability value of 1 was selected as optimal, as it has all the mentioned properties. The software provided both actual and predicted values for the optimized formulation’s dependent variable (responses). The optimized formulation independent variable values were as follows: stirring speed (X1) was 600 RPM, surfactant concentration (X2) was 1%, and polymer concentration (X3) was 400 mg.

The optimized formulation predicted response values were as follows: particle size (Y1) 228.9 nm, PDI (Y2) 0.3, and %EE (Y3) 72.75%. Upon preparing optimized cinnamon-loaded Eudragit nanoparticles using the values predicted by the software, the ascertained dependent variable (response) values were aligned closely with predicted values. The actual values of optimized cinnamon EO-loaded Eudragit nanoparticles were as follows: particle size (Y1) was 230.4 ± 3.46 nm, PDI (Y2) was 0.293 ± 0.022, and %EE (Y3) was 74.9% ± 2.32%. Moreover, the zeta potential of the optimized formulation was recorded as −23 mV.

The close correspondence of the predicted and actual values confirms the reliability and accuracy of the optimization process. The optimized cinnamon-loaded Eudragit nanoparticles were subjected to further characterization studies, including an *in vitro* release study followed by kinetic modelling.

### 3.6 *In vitro* drug release

**FIGURE 7:**
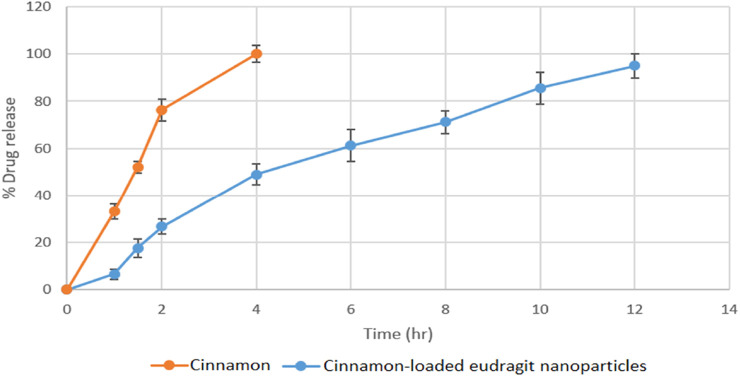
*In vitro* release profile of cinnamon and cinnamon-loaded Eudragit nanoparticles.

**FIGURE 8:**
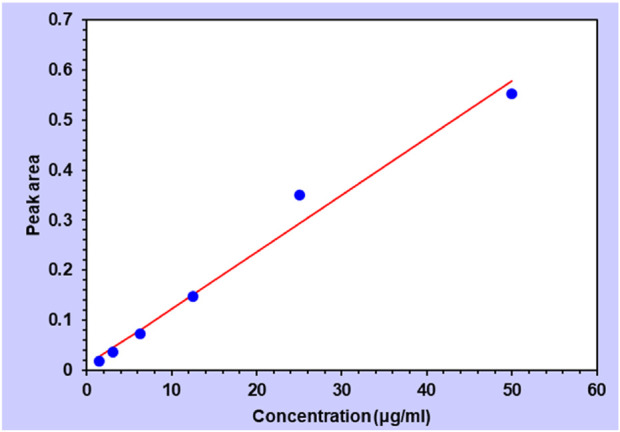
Standard curve of the cinnamon EO (*R* ^2^ value 0.981).

The optimal drug carrier should be able to enhance the binding efficiency and extend the release duration. The drug delivery system must have the capacity to contain an adequate amount of the active compound to ensure its extended release at a targeted site, thereby decreasing both frequency and dosage of administration. Figures 7, 8 represents the release profile of cinnamon-loaded Eudragit nanoparticles. These profiles show that cinnamon release from engineered nanoparticles was more sustained than free cinnamon over a 12-h period. Especially, the absence of an initial burst release shows that cinnamon was encapsulated and engrafted within Eudragit L100 rather than barely adsorbing on the polymer surface. These observation findings were aligned with previous research findings (Table 8).

**TABLE 8:** Different kinetic models applied to optimized cinnamon-loaded nanoparticle release data.

Models	*R* ^2^	R-adjust	SS	MSC
Zero-order	0.9495	0.9495	488.86	2.51
First-order	0.9792	0.9792	201.66	3.404
Korsmeyer–Peppas	0.9857	0.9836	138.71	3.56
Higuchi	0.9284	0.9284	693.84	2.17

The kinetic analysis of cinnamon release from optimized cinnamon-loaded Eudragit nanoparticles was conducted. The release data of the optimized formulation did not align well with zero-order, first-order, and Higuchi models. Fitting the release data of optimized formulation to the Peppas model provided a more precise and accurate representation of drug release kinetics. The data were analyzed using the Peppas model, and an n value for optimized cinnamon-loaded Eudragit nanoparticles was determined. The n value of the formulation was 0.74, which indicates non-Fickian and anomalous drug release behavior of the formulation affected by multiple mechanisms, including polymer relaxation, diffusion, and erosion.

### 3.7 FTIR analysis

**FIGURE 9:**
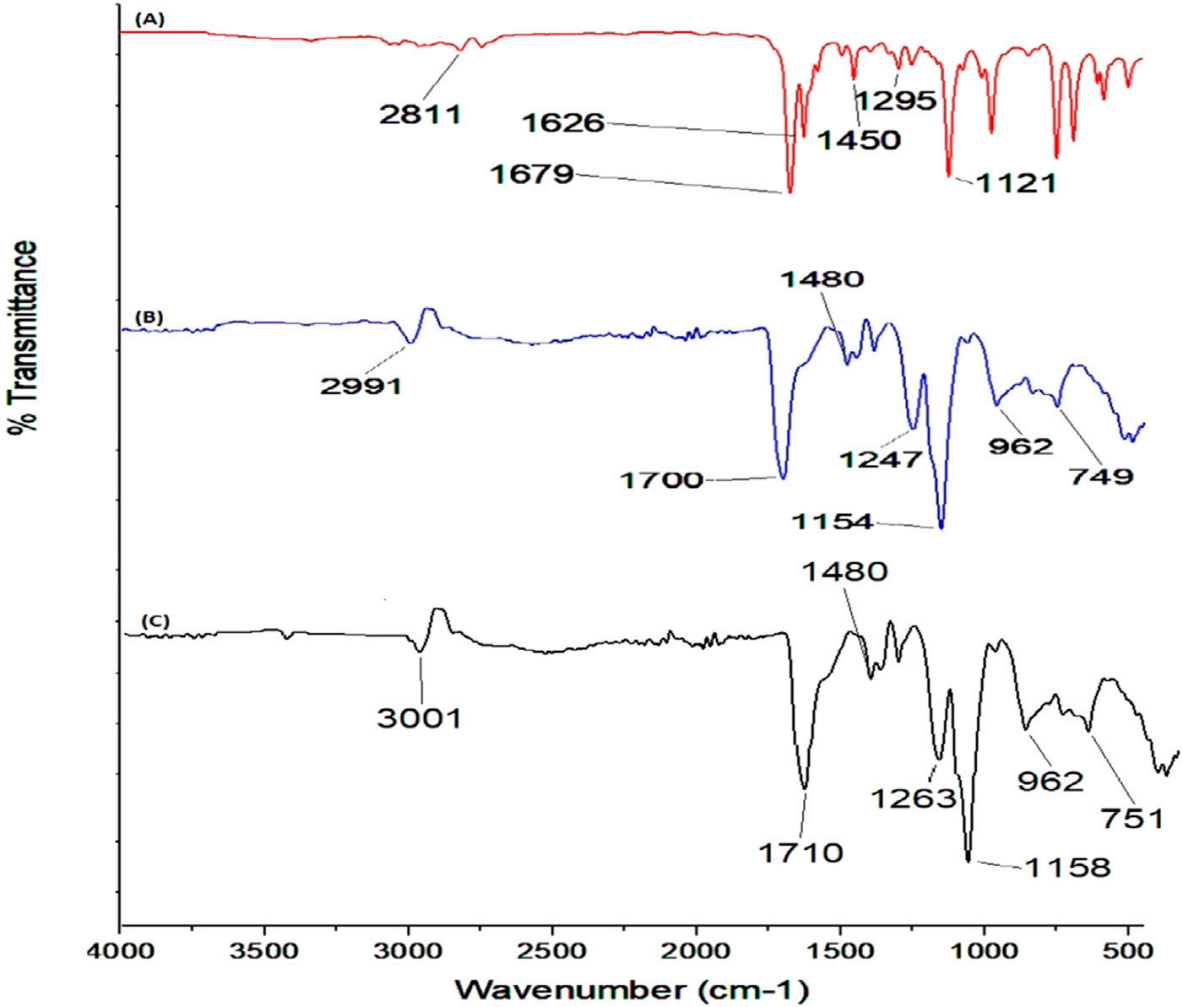
FTIR spectra of **(A)** cinnamon essential oil, **(B)** Eudragit L100, and **(C)** cinnamon essential oil-loaded nanoparticles.

FTIR was performed to check for any type of interaction between the active ingredients and the polymer used in the formulation of the nanoparticles. The FTIR spectrum of cinnamon EO (A) is shown in Figure 8, where the peak at 1,679 cm^−1^ represents the stretching vibration of the carbonyl group (C=O), while those at 1,626 cm^−1^, 1,450 cm^−1^, and 1,295 cm^−1^ represent the stretching vibrations of the C=C, OH of the aromatic ring (Ullah et al., 2022; Yang et al., 2021). The FTIR spectrum of Eudragit L100 displayed several characteristic peaks at 1,700 cm^−1^ (C O carboxylic acid group vibrations), 1738 cm^−1^ (esterified carboxyl group vibrations), 1,159 cm^−1^, 1,180 cm^−1^, and 1,263 cm^−1^ (ester vibrations), 1,388 cm^−1^, 1,475 cm^−1^, and 2,989 cm^−1^ (CHX vibrations), and 3,235 cm^−1^ (OH groups vibrations) (Figure 9) (Hao et al., 2013). There was no new band in the IR spectra of the cinnamon EO-loaded Eudragit L100 nanoparticles, but it appeared to be a combination of the essential oil and polymer, indicating no major chemical interaction of the cinnamon EO with the nanoparticle.

### 3.8 Surface morphology

**FIGURE 10:**
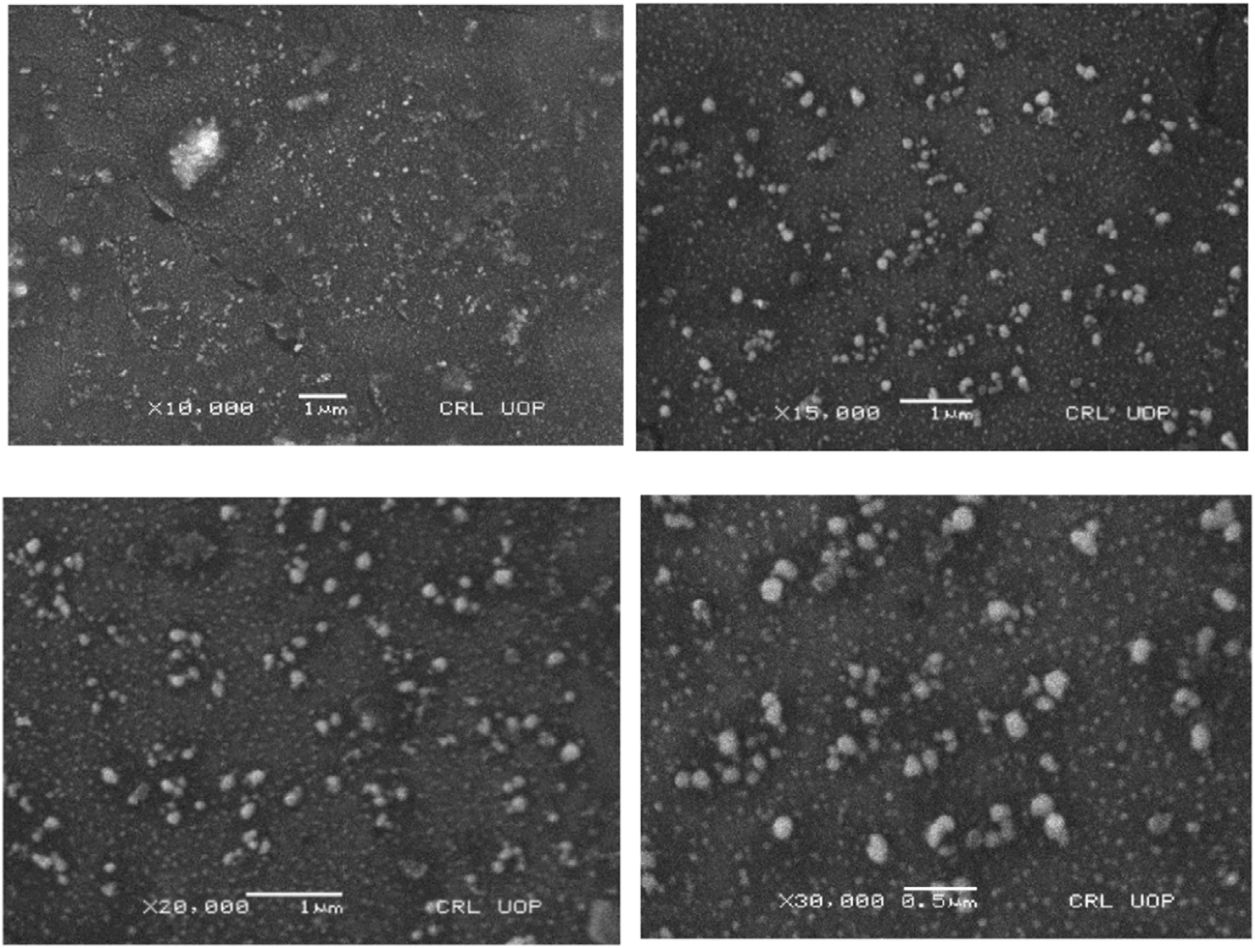
Zeta potential of the optimized formulation.

A scanning electron microscope was used to conduct a morphological analysis of cinnamon-loaded Eudragit nanoparticles synthesized via the nanoprecipitation technique. The SEM examination provided comprehensive details of particle shape, surface texture, overall surface smoothness, and inter-particulate bridging of cinnamon-loaded Eudragit nanoparticles (Figure 10). This particle smoothness indicates a surface structure advantageous to controlled cinnamon release, likely facilitated by diffusion, matrix erosion, and polymer chain relaxation. Additionally, the SEM images also show visible bridging between particles, which may be ascribed to the adhesive properties of the polyvinyl acetate (PVA) used in formulation preparation. Due to the inherent stickiness properties of the PVA, complete removal of PVA from cinnamon-loaded Eudragit nanoparticles proved arduous even after extensive washing.

### 3.9 Effect of zeta potential

**FIGURE 11:**
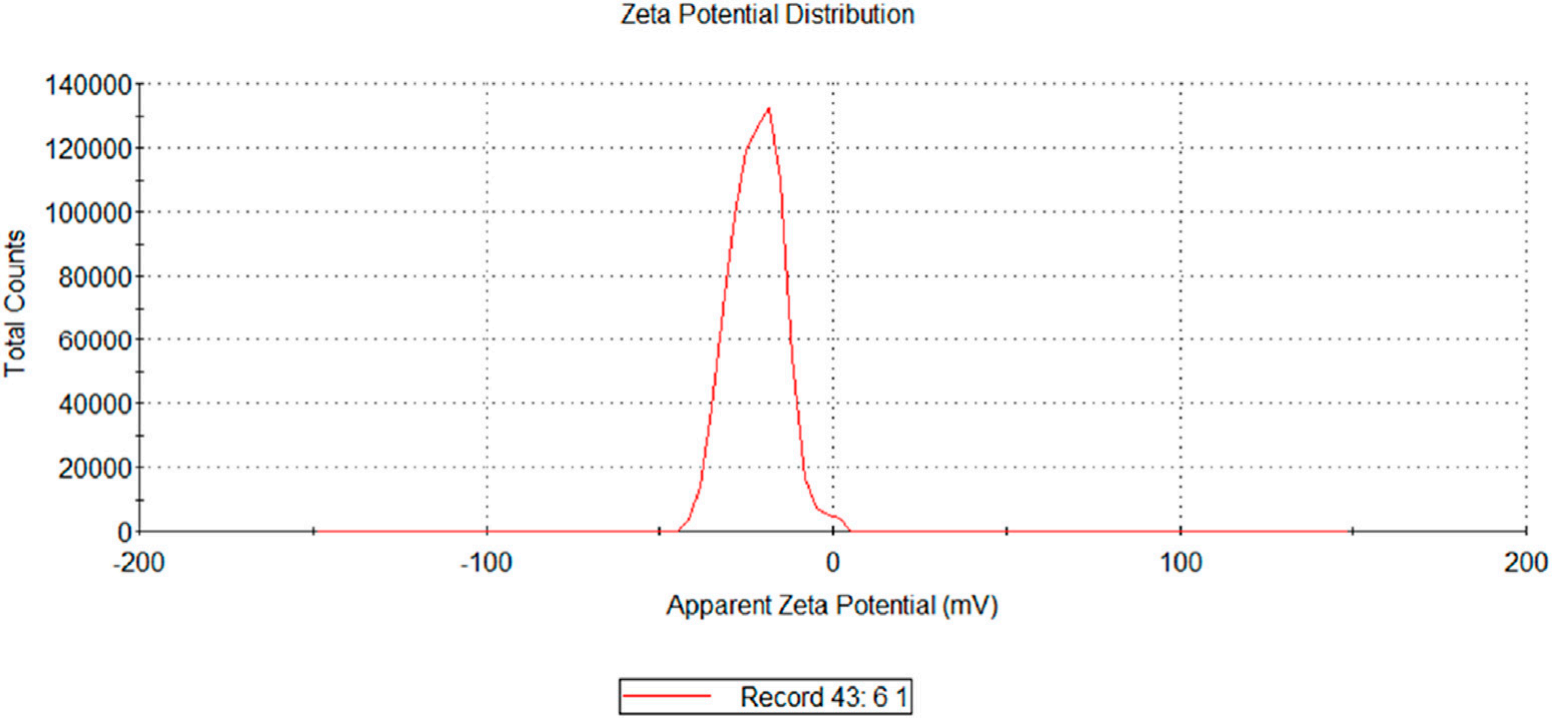
Graph of the average size of the optimized formulation of nanoparticles.

**FIGURE 12:**
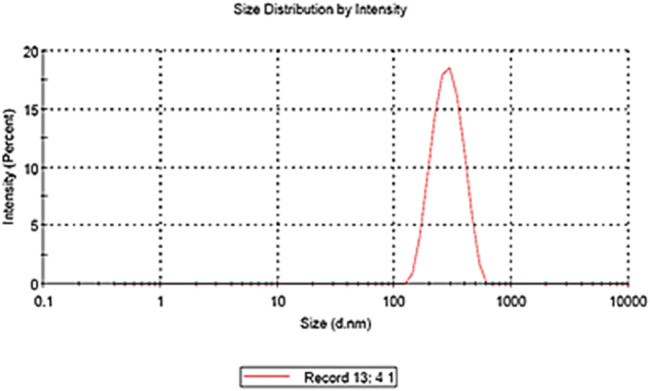
Scanning electron microscopy of the cinnamon-loaded Eudragit L100-based nanoparticles.

Zeta potential measures the electrical potential at the shear plane between the surface of a particle and the surrounding liquid (Lin et al., 2024). High zeta potential (positive or negative) indicates strong electrostatic repulsion between particles, promoting stability and preventing aggregation. A low zeta potential, conversely, suggests weaker repulsion and a greater tendency for particles to clump together (Doostmohammadi et al., 2011). The zeta potential values of the 16 formulations range from −17.4 mV to −55.6 mV, indicating that all formulations carry a negative surface charge (Figures 11, 12). Most values lie between approximately −17 mV and −27 mV, which suggests moderate electrostatic stability, meaning the formulations are likely to have reasonable dispersion stability due to sufficient repulsive forces preventing particle aggregation (Jiang et al., 2009; Shrestha et al., 2020). Notably, two formulations show significantly higher negative values, at −55.6 mV and −44.6 mV, indicating much stronger surface charge and thus enhanced colloidal stability (Zubir et al., 2015). These formulations are expected to have superior resistance to aggregation and better suspension stability than others (Prakash et al., 2014). Overall, the negative zeta potential across all formulations suggests a favorable electrostatic environment for maintaining dispersion, with the formulations exhibiting stronger negative charges potentially being the most stable (Arshad et al., 2024; Kamble et al., 2022). The predicted zeta potential value of −26.0 mV generated by the software closely aligns with the experimentally calculated value of −23.0 ± 1 mV (Figures 11, 12). This close agreement indicates that the predictive model reliably estimates the surface charge characteristics of the formulation. The minor difference between the predicted and measured values may arise from experimental variability, sample heterogeneity, or limitations in the model’s assumptions.

### 3.10 Antimicrobial evaluation

**FIGURE 13:**
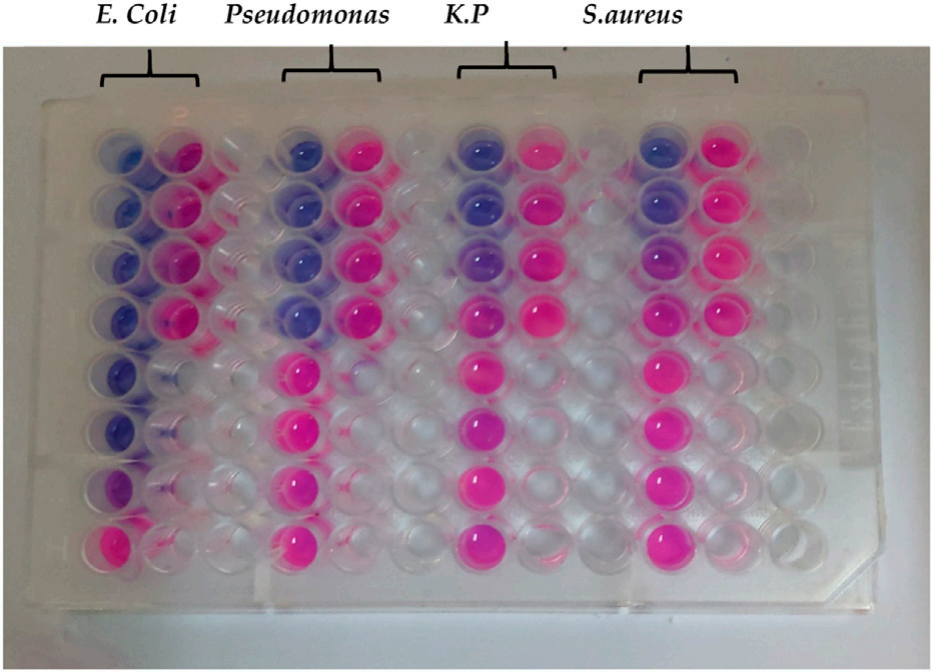
Minimum inhibitory concentration of the cinnamon essential oil against different resistant bacterial strains.

**FIGURE 14:**
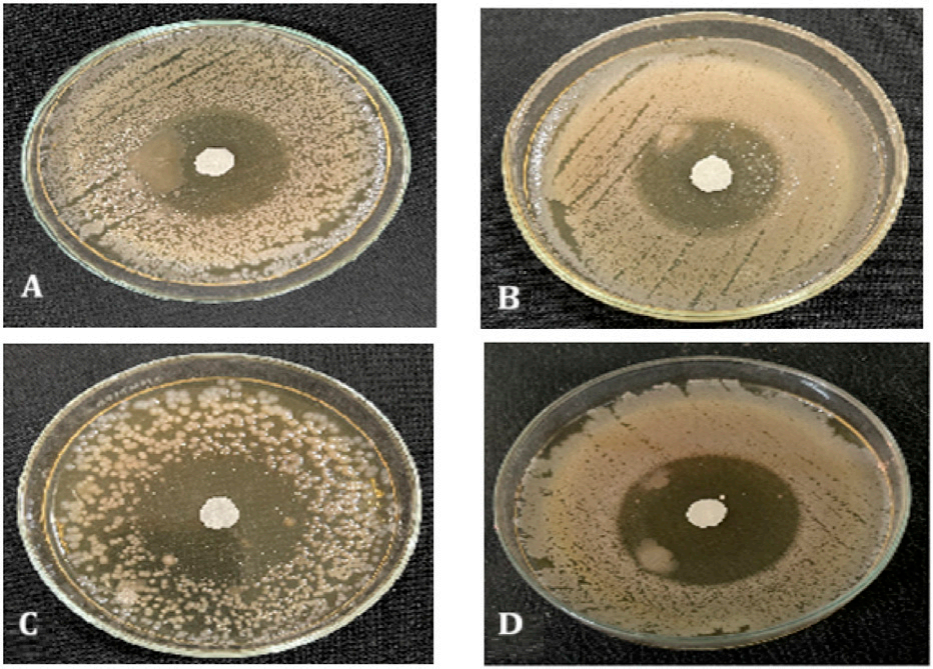
Zone of inhibition of nano formulation against **(A)** *Klebsiella pneumoniae*, **(B)** *Staphylococcus aureus*, **(C)** *Pseudomonas aeruginosa*, and **(D)** *E. coli*.

The minimum inhibitory concentration of the cinnamon essential oil was determined using a broth dilution method on a 96-well microplate. It was observed that the MIC of the cinnamon EO against *E. coli* was 0.078 μL/mL and against *Pseudomonas aeruginosa* it was 0.063 μL/mL, while against *Klebsiella pneumoniae* and *Staphylococcus aureus*, it was the same, at 1.25 μL/mL (Table 9; Figure 13). On the other hand, the antimicrobial activity of prepared formulations (cinnamon essential oil loaded) was determined against *S. aureus, Klebsiella,* and *Pseudomonas aeruginosa*, and it was observed that cinnamon essential oil exhibited antibacterial properties. The largest inhibition zones (17 mm) was shown against the *E. coli* (Table 10; Figure 14). The prepared cinnamon EO-loaded nanoparticles showed more efficacy against Gram-negative bacteria than against Gram-positive bacteria, as reported earlier (Vahedikia et al., 2019). The enhanced antimicrobial effect of the cinnamon essential oil-based nanoemulgel can be attributed to the increased surface area and bioavailability of the essential oil, which facilitates better penetration and interaction with microbial membranes (Liu et al., 2014; Jamir et al., 2024). The nanoemulsion’s small particle size enables a sustained release of active compounds, leading to prolonged antimicrobial activity at the target site (Wang et al., 2021). Additionally, the encapsulation of cinnamon oil within the nanoemulgel matrix protects the active ingredients from degradation, maintaining their potency and improving the overall effectiveness of the formulation (Hosny et al., 2021). Cinnamon EO contains cinnamaldehyde, which disrupts bacterial cell membranes, interferes with ATP synthesis, and inhibits quorum sensing (Shu et al., 2024).

**TABLE 9:** Minimum inhibitory concentration of cinnamon essential oil.

Essential oil	Minimum inhibitory concentration (µL/mL)			
	*E. coli*	*Pseudomonas*	*K. P*	*S. aureus*
Cinnamon essential oil	0.078	0.063	1.25	1.25

**TABLE 10:** Zone of inhibition of the cinnamon essential oil-loaded formulation against different bacterial strains.

S/No	Bacterial strain	Zone of inhibition (mm)
1	*E.coli*	17
2	*Klebsiella pneumoniae*	13
4	*Styphylococcus aureus*	10
5	*Pseudomonas aeruginosa*	15

Polymers in nanoplatforms play a crucial role in enhancing the efficacy by improving the stability, controlled release, and bioavailability of active compounds. Chitosan and PLGA-based formulations for cinnamon essential oil (CEO) encapsulation show improved stability, controlled release, and antimicrobial effects compared to non-encapsulated oils (Ghaderi-Ghahfarokhi et al., 2017; Hashim et al., 2024). Additionally, they have inherent antimicrobial properties (Ignjatović et al., 2016) that can further contribute to the overall antimicrobial activity of the nanoplatforms and enhance the treatment’s effectiveness against pathogens (Li et al., 2017). However, Eudragit-based formulations provide better encapsulation efficiency and stability due to their enhanced barrier properties, preventing degradation of volatile bioactive compounds like cinnamaldehyde (Patra et al., 2017; Feng et al., 2022). Unlike chitosan or PLGA, Eudragit is more resistant to environmental factors, leading to prolonged release and sustained antimicrobial activity over time (Yurtdaş-Kırımlıoğlu et al., 2020; da Silva et al., 2024). Therefore, Eudragit-based nanoencapsulation offers the best protection and effectiveness for cinnamon essential oil, making it the ideal choice for enhancing both stability and bioavailability.

## 4 Conclusion

This study reports the development of Eudragit-based nanoparticles doped with cinnamon essential oil with the aim of eradicating resistant bacterial strains. Cinnamon essential oil-incorporated Eudragit nanoparticles show a significant zone of inhibition and MIC value against various resistant bacterial strains like *E. coli, Klebsiella, Staphylococcus aureus*, and *Pseudomonas aeruginosa.* The different formulations were developed and optimized with Design-Expert software on the basis of particle size, PDI, and entrapment efficiency. Vibrational analysis and surface morphology revealed that nanoparticles show no interaction with active and inactive moieties and are properly doped with cinnamon essential oil, with a smooth and uniform appearance of the formulation. The encapsulation of cinnamon essential oil in nanoparticles leads to more sustained release compared to free cinnamon oil over a 12-h period. Hence, Eudragit-based nanoparticles incorporated with CEO are advocated to be a promising platform for the treatment of resistant bacterial strains.

## Data Availability

The original contributions presented in the study are included in the article/supplementary material; further inquiries can be directed to the corresponding author.

## References

[B1] Al Zuhairi J. J. M. J. Kashi F. J. Rahimi-Moghaddam A. Yazdani M. (2020). Antioxidant, cytotoxic and antibacterial activity of *Rosmarinus officinalis* L. essential oil against bacteria isolated from urinary tract infection. Eur. J. Integr. Med. 38, 101192. 10.1016/j.eujim.2020.101192

[B2] Arshad A. Arshad S. Mahmood A. Asim M. H. Ijaz M. Irfan H. M. (2024). Zeta potential changing self-nanoemulsifying drug delivery systems: a newfangled approach for enhancing oral bioavailability of poorly soluble drugs. Int. J. Pharm. 655, 123998. 10.1016/j.ijpharm.2024.123998 38490401

[B3] Baekkeskov E. Rubin O. Munkholm L. Zaman W. (2020). Antimicrobial resistance as a global health crisis. Oxf. Res. Encycl. Polit. 1–24. 10.1093/acrefore/9780190228637.013.1626

[B4] Bin-Jumah M. Gilani S. J. Jahangir M. A. Zafar A. Alshehri S. Yasir M. (2020). Clarithromycin-loaded ocular chitosan nanoparticle: formulation, optimization, characterization, ocular irritation, and antimicrobial activity. Int. J. Nanomedicine 15, 7861–7875. 10.2147/ijn.s269004 33116505 PMC7568680

[B5] Chakansin C. Yostaworakul J. Warin C. Kulthong K. Boonrungsiman S. (2022). Resazurin rapid screening for antibacterial activities of organic and inorganic nanoparticles: potential, limitations and precautions. Anal. Biochem. 637, 114449. 10.1016/j.ab.2021.114449 34762874

[B6] Chinemerem Nwobodo D. Ugwu M. C. Oliseloke Anie C. Al-Ouqaili M. T. S. Chinedu Ikem J. Victor Chigozie U. (2022). Antibiotic resistance: the challenges and some emerging strategies for tackling a global menace. J. Clin. Laboratory Analysis 36 (9), e24655–10. 10.1002/jcla.24655 PMC9459344 35949048

[B71] da Silva A. B. Facchi S. P. Bezerra F. M. Lis M. J. Monteiro J. P. Bonafé E. G. (2024). Antimicrobial composites based on methacrylic acid–methyl methacrylate electrospun fibers stabilized with copper (II). Molecules 29 (12), 2835. 38930901 10.3390/molecules29122835 PMC11206514

[B7] Dadgostar P. (2019). Antimicrobial resistance: implications and costs. Infect. Drug Resist. 12, 3903–3910. 10.2147/IDR.S234610 31908502 PMC6929930

[B8] Dghais S. Ben Jemaa M. Chouchen M. Jallouli S. Ksouri R. Falleh H. (2023). Nano-emulsification of cinnamon and curcuma essential oils for the quality improvement of minced meat beef. Foods 12 (2), 235–10. 10.3390/foods12020235 36673327 PMC9857730

[B9] Di Leo G. Sardanelli F. (2020). Statistical significance: p value, 0.05 threshold, and applications to radiomics—reasons for a conservative approach. Eur. Radiol. Exp. 4, 18–8. 10.1186/s41747-020-0145-y 32157489 PMC7064671

[B10] Doostmohammadi A. Monshi A. Salehi R. Fathi M. H. Golniya Z. Daniels A. U. (2011). Bioactive glass nanoparticles with negative zeta potential. Ceram. Int. 37 (7), 2311–2316. 10.1016/j.ceramint.2011.03.026

[B11] Dos Santos J. da Silva G. S. Velho M. C. Beck R. C. R. (2021). Eudragit®: a versatile family of polymers for hot melt extrusion and 3D printing processes in pharmaceutics. Pharmaceutics 13 (9), 1424–1436. 10.3390/pharmaceutics13091424 34575500 PMC8471576

[B12] El Atki Y. Aouam I. El Kamari F. Taroq A. Nayme K. Timinouni M. (2019). Antibacterial activity of cinnamon essential oils and their synergistic potential with antibiotics. J. Adv. Pharm. Technol. Res. 10 (2), 63–67. 10.4103/japtr.JAPTR_366_18 31041184 PMC6474160

[B13] Elmowafy M. Alhakamy N. A. Shalaby K. Alshehri S. Ali H. M. Mohammed E. F. (2021). Hybrid polylactic acid/Eudragit L100 nanoparticles: a promising system for enhancement of bioavailability and pharmacodynamic efficacy of luteolin. J. Drug Deliv. Sci. Technol. 65, 102727. 10.1016/j.jddst.2021.102727

[B69] Feng S. Bandari S. Repka M. A. (2022). Investigation of poly (2-ethyl-2-oxazoline) as a novel extended release polymer for hot-melt extrusion paired with fused deposition modeling 3D printing. J. Drug. Deliv. Sci. Technol. 74, 103558.

[B63] Ghaderi-Ghahfarokhi M. Barzegar M. Sahari M. A. Gavlighi H. A. Gardini F. (2017). Chitosan-cinnamon essential oil nano-formulation: application as a novel additive for controlled release and shelf life extension of beef patties. Int. J. Biol. Macromol. 102, 19–28. 28380334 10.1016/j.ijbiomac.2017.04.002

[B16] Hadidi M. Pouramin S. Adinepour F. Haghani S. Jafari S. M. (2020). Chitosan nanoparticles loaded with clove essential oil: characterization, antioxidant and antibacterial activities. Carbohydr. Polym. 236, 116075. 10.1016/j.carbpol.2020.116075 32172888

[B17] Hao S. Wang B. Wang Y. Zhu L. Wang B. Guo T. (2013). Preparation of Eudragit L 100-55 enteric nanoparticles by a novel emulsion diffusion method. Colloids Surfaces B Biointerfaces 108, 127–133. 10.1016/j.colsurfb.2013.02.036 23537830

[B64] Hashim L. E. Sabri A. H. Mohamad M. A. Anjani Q. K. Mustaffa M. F. Abdul Hamid K. (2024). Circumventing the gastrointestinal barrier for oral delivery of therapeutic proteins and peptides (PPTs): current trends and future trajectories. Curr. Drug Deliv. 21 (2), 211–235. 37076462 10.2174/1567201820666230418091506

[B60] Hosny K. M. Khallaf R. A. Asfour H. Z. Rizg W. Y. Alhakamy N. A. Sindi A. M. (2021). Development and optimization of cinnamon oil nanoemulgel for enhancement of solubility and evaluation of antibacterial, antifungal and analgesic effects against oral microbiota. Pharmaceutics 13 (7), 1008. 34371700 10.3390/pharmaceutics13071008 PMC8309164

[B65] Ignjatović N. Wu V. Ajduković Z. Mihajilov-Krstev T. Uskoković V. Uskoković D. (2016). Chitosan-PLGA polymer blends as coatings for hydroxyapatite nanoparticles and their effect on antimicrobial properties, osteoconductivity and regeneration of osseous tissues. Mater. Sci. Eng. C 60, 357–364. 10.1016/j.msec.2015.11.061 PMC4780868 26706541

[B18] Imran M. Shah A. H. Ullah N. Alomar S. Y. Rehman A. Rehman N. U. (2024). Integrated computational analysis, *in vitro*, *in vivo* investigation on Myristica fragrans Houtt. essential oils for potential anti rheumatic activities. J. King Saud University-Science 36 (5), 103177. 10.1016/j.jksus.2024.103177

[B61] Jamir Y. Bhushan M. Sanjukta R. Robindro Singh L. (2024). Plant‐based essential oil encapsulated in nanoemulsions and their enhanced therapeutic applications: an overview. Biotechnol. Bioeng. 121 (2), 415–433. 37941510 10.1002/bit.28590

[B19] Jiang J. Oberdörster G. Biswas P. (2009). Characterization of size, surface charge, and agglomeration state of nanoparticle dispersions for toxicological studies. J. Nanoparticle Res. 11, 77–89. 10.1007/s11051-008-9446-4

[B20] Jummes B. Sganzerla W. G. da Rosa C. G. Noronha C. M. Nunes M. R. Bertoldi F. C. (2020). Antioxidant and antimicrobial poly-ε-caprolactone nanoparticles loaded with Cymbopogon martinii essential oil. Biocatal. Agric. Biotechnol. 23, 101499. 10.1016/j.bcab.2020.101499

[B21] Kacaniova M. Galovicova L. Valkova V. Tvrda E. Terentjeva M. Ziarovska J. (2021). Antimicrobial and antioxidant activities of Cinnamomum cassia essential oil and its application in food preservation. Open Chem. 19 (1), 214–227. 10.1515/chem-2021-0191

[B22] Kamble S. Agrawal S. Cherumukkil S. Sharma V. Jasra R. V. Munshi P. (2022). Revisiting zeta potential, the key feature of interfacial phenomena, with applications and recent advancements. ChemistrySelect 7 (1), e202103084. 10.1002/slct.202103084

[B23] Kiparissides C. Pladis P. (2022). On the prediction of suspension viscosity, grain morphology, and agitation power in SPVC reactors. Can. J. Chem. Eng. 100 (4), 714–730. 10.1002/cjce.24262

[B24] Knauth P. López Z. L. Hernández G. J. A. Sevilla M. T. E. (2018). “Cinnamon essential oil: chemical composition and biological activities,” in Essential oils production, applications and health benefits.

[B25] Kumar M. Hilles A. R. Almurisi S. H. Bhatia A. Mahmood S. (2023). Micro and nano-carriers-based pulmonary drug delivery system: their current updates, challenges, and limitations – a review. JCIS Open 12 (May), 100095. 10.1016/j.jciso.2023.100095

[B26] Lammari N. Louaer O. Meniai A. H. Elaissari A. (2020). Encapsulation of essential oils via nanoprecipitation process: overview, progress, challenges and prospects. Pharmaceutics 12 (5), 431–21. 10.3390/pharmaceutics12050431 32392726 PMC7284627

[B27] Lee M. H. Park H. J. (2015). Preparation of halloysite nanotubes coated with Eudragit for a controlled release of thyme essential oil. J. Appl. Polym. Sci. 42771, 1–7. 10.1002/app.42771

[B67] Li Y. Na R. Wang X. Liu H. Zhao L. Sun X. (2017). Fabrication of antimicrobial peptide-loaded PLGA/chitosan composite microspheres for long-acting bacterial resistance. Molecules 22 (10), 1637. 28961197 10.3390/molecules22101637 PMC6151433

[B28] Liao W. Badri W. Dumas E. Ghnimi S. Elaissari A. Saurel R. (2021). Nanoencapsulation of essential oils as natural food antimicrobial agents: an overview. Appl. Sci. Switz. 11 (13), 5778. 10.3390/app11135778

[B29] Lin S. Li J. Hu X. Chen S. Huang H. Wu Y. (2024). Potential dietary calcium supplement: calcium-chelating peptides and peptide-calcium complexes derived from blue food proteins. Trends Food Sci. Technol. 145, 104364. 10.1016/j.tifs.2024.104364

[B30] Liu W. L. Xiao Z. B. Zhu G. Y. Zhou R. J. Wang E. Q. Niu Y. W. (2014). Production and properties of mononuclear microcapsules encapsulating cinnamon oil by complex coacervation. Appl. Mech. Mater. 477–478, 1229–1233. 10.4028/www.scientific.net/AMM.477-478.1229

[B31] Malik S. Muhammad K. Waheed Y. (2023). Nanotechnology: a revolution in modern industry. Molecules 28 (2), 661. 10.3390/molecules28020661 36677717 PMC9865684

[B32] Manisekaran A. Grysan P. Duez B. Schmidt D. F. Lenoble D. Thomann J.-S. (2022). Solvents drive self-assembly mechanisms and inherent properties of Kraft lignin nanoparticles (<50 nm). J. Colloid Interface Sci. 626, 178–192. 10.1016/j.jcis.2022.06.089 35785603

[B33] Mohsen A. M. Nagy Y. I. Shehabeldine A. M. Okba M. M. (2023). Thymol-loaded eudragit RS30D cationic nanoparticles-based hydrogels for topical application in wounds: *in vitro* and *in vivo* evaluation. Pharmaceutics 15 (1), 19. 10.3390/pharmaceutics15010019 PMC9861126 36678648

[B34] Nawaz T. Iqbal M. Khan B. A. Nawaz A. Hussain T. Hosny K. M. (2021). Development and optimization of acriflavine-loaded polycaprolactone nanoparticles using box–behnken design for burn wound healing applications. Polymers 14 (1), 101. 10.3390/polym14010101 35012125 PMC8747314

[B35] Niu B. Yan Z. Shao P. Kang J. Chen H. (2018). Encapsulation of cinnamon essential oil for active food packaging film with synergistic antimicrobial activity. Nanomaterials 8 (8), 598. 10.3390/nano8080598 30082645 PMC6116257

[B36] Onugwu A. L. Attama A. A. Nnamani P. O. Onugwu S. O. Onuigbo E. B. Khutoryanskiy V. V. (2022). Development and optimization of solid lipid nanoparticles coated with chitosan and poly(2-ethyl-2-oxazoline) for ocular drug delivery of ciprofloxacin. J. Drug Deliv. Sci. Technol. 74, 103527. 10.1016/j.jddst.2022.103527

[B68] Patra C. N. Priya R. Swain S. Jena G. K. Panigrahi K. C. Ghose D. (2017). Pharmaceutical significance of Eudragit: A review. Future J. Pharm. Sci. 3 (1), 33–45.

[B37] Prakash S. Mishra R. Malviya R. Sharma P. K. (2014). Measurement techniques and pharmaceutical applications of zeta potential: a review. J. Chronotherapy Drug Deliv. 5 (2), 33–40.

[B38] Routray A. Senapati P. K. Padhy M. Das D. (2022). Effect of mixture of natural and synthetic surfactant and particle size distribution for stabilized high-concentrated coal water slurry. Int. J. Coal Prep. Util. 42, 238–253. 10.1080/19392699.2019.1592166

[B39] Ruiz E. Orozco V. H. Hoyos L. M. Giraldo L. F. (2022). Study of sonication parameters on PLA nanoparticles preparation by simple emulsion-evaporation solvent technique. Eur. Polym. J. 173, 111307. 10.1016/j.eurpolymj.2022.111307

[B40] Saha M. Sarkar A. (2021). Review on multiple facets of drug resistance: a rising challenge in the 21st century. J. Xenobiotics 11 (4), 197–214. 10.3390/jox11040013 PMC8708150 34940513

[B41] Satchanska G. Davidova S. Petrov P. D. (2024). Natural and synthetic polymers for biomedical and environmental applications. Polymers 16 (8), 1159. 10.3390/polym16081159 38675078 PMC11055061

[B42] Schwaminger S. P. Syhr C. Berensmeier S. (2020). Controlled synthesis of magnetic iron oxide nanoparticles: magnetite or maghemite? Crystals 10 (3), 214. 10.3390/cryst10030214

[B43] Sharma B. (2021). Superbugs: the nightmare bacteria. J. Animal Res. 11 (5). 10.30954/2277-940x.05.2021.1

[B44] Sharma N. Madan P. Lin S. (2016). Effect of process and formulation variables on the preparation of parenteral paclitaxel-loaded biodegradable polymeric nanoparticles: a co-surfactant study. Asian J. Pharm. Sci. 11 (3), 404–416. 10.1016/j.ajps.2015.09.004

[B45] Shrestha S. Wang B. Dutta P. (2020). Nanoparticle processing: understanding and controlling aggregation. Adv. Colloid Interface Sci. 279, 102162. 10.1016/j.cis.2020.102162 32334131

[B46] Shu C. Ge L. Li Z. Chen B. Liao S. Lu L. (2024). Antibacterial activity of cinnamon essential oil and its main component of cinnamaldehyde and the underlying mechanism. Front. Pharmacol. 15, 1378434. 10.3389/fphar.2024.1378434 38529191 PMC10961361

[B47] Siagian H. Tarigan Z. J. H. Jie F. (2021). Supply chain integration enables resilience, flexibility, and innovation to improve business performance in COVID-19 era. Sustainability 13 (9), 4669. 10.3390/su13094669

[B48] Spence C. (2024). Cinnamon: the historic spice, medicinal uses, and flavour chemistry. Int. J. Gastron. Food Sci. 35 (November 2023), 100858. 10.1016/j.ijgfs.2023.100858

[B49] Subasinghe U. G. P. P. Wickramarachchi S. (2019). Encapsulation of cinnamon leaf oil within chitosan: formulation and characterization. Ceylon J. Sci. 48 (3), 279. 10.4038/cjs.v48i3.7652

[B50] Trucillo P. (2024). Biomaterials for drug delivery and human applications. Materials 17 (2), 456. 10.3390/ma17020456 38255624 PMC10817481

[B51] Ullah A. Khan N. R. Khan M. H. Mehmood S. Khan J. Iftikhar T. (2021). Formulation of microwave-assisted natural-synthetic polymer composite film and its physicochemical characterization. Int. J. Polym. Sci. 2021, 1–13. 10.1155/2021/9961710

[B52] Ullah N. Amin A. Alamoudi R. A. Rasheed S. A. Alamoudi R. A. Nawaz A. (2022). Fabrication and optimization of essential-oil-loaded nanoemulsion using box–behnken design against Staphylococos aureus and Staphylococos epidermidis isolated from oral cavity. Pharmaceutics 14 (8), 1640. 10.3390/pharmaceutics14081640 36015266 PMC9416493

[B53] Ullah N. Amin A. Farid A. Selim S. Rashid S. A. Aziz M. I. (2023). Development and evaluation of essential oil-based nanoemulgel formulation for the treatment of oral bacterial infections. Gels 9 (3), 252. 10.3390/gels9030252 36975701 PMC10048686

[B54] Vahedikia N. Garavand F. Tajeddin B. Cacciotti I. Jafari S. M. Omidi T. (2019). Biodegradable zein film composites reinforced with chitosan nanoparticles and cinnamon essential oil: physical, mechanical, structural and antimicrobial attributes. Colloids Surfaces B Biointerfaces 177, 25–32. 10.1016/j.colsurfb.2019.01.045 30703751

[B62] Wang Y. Cen C. Chen J. Zhou C. Fu L. (2021). Nano-emulsification improves physical properties and bioactivities of litsea cubeba essential oil. LWT 137, 110361.

[B55] Weisany W. Yousefi S. Tahir N. A. razzak Golestanehzadeh N. McClements D. J. Adhikari B. (2022). Targeted delivery and controlled released of essential oils using nanoencapsulation: a review. Adv. Colloid Interface Sci. 303 (March), 102655. 10.1016/j.cis.2022.102655 35364434

[B57] Yang K. Liu A. Hu A. Li J. Zen Z. Liu Y. (2021). Preparation and characterization of cinnamon essential oil nanocapsules and comparison of volatile components and antibacterial ability of cinnamon essential oil before and after encapsulation. Food Control. 123, 107783. 10.1016/j.foodcont.2020.107783

[B70] Yurtdaş-Kırımlıoğlu G. Görgülü Ş. Berkman M. S. (2020). Novel approaches to cancer therapy with ibuprofen-loaded Eudragit® RS 100 and/or octadecylamine-modified PLGA nanoparticles by assessment of their effects on apoptosis. Drug Dev. Ind. Pharm. 46 (7), 1133–1149. 32476502 10.1080/03639045.2020.1776319

[B58] Yusuf A. Almotairy A. R. Z. Henidi H. Alshehri O. Y. Aldughaim M. S. (2023). Nanoparticles as drug delivery systems: a review of the implication of nanoparticles’ physicochemical properties on responses in biological systems. Polymers 15 (7), 1596. 10.3390/polym15071596 37050210 PMC10096782

[B59] Zubir M. N. M. Badarudin A. Kazi S. N. Misran M. Amiri A. Sadri R. (2015). Experimental investigation on the use of highly charged nanoparticles to improve the stability of weakly charged colloidal system. J. Colloid Interface Sci. 454, 245–255. 10.1016/j.jcis.2015.05.019 26048724

